# Selection of complementary foods based on optimal nutritional values

**DOI:** 10.1038/s41598-017-05650-0

**Published:** 2017-07-14

**Authors:** Partho Sen, Adil Mardinogulu, Jens Nielsen

**Affiliations:** 1 Department of Biology and Biological Engineering, Chalmers University of Technology, Kemivägen 10, SE-412 96 Göteborg, Sweden; 2 Science for Life Laboratory, KTH - Royal Institute of Technology, SE-171 21 Stockholm, Sweden; 3 Novo Nordisk Foundation Center for Biosustainability, Technical University of Denmark, DK2800 Lyngby, Denmark

## Abstract

Human milk is beneficial for growth and development of infants. Several factors result in mothers ceasing breastfeeding which leads to introduction of breast-milk substitutes (BMS). In some communities traditional foods are given as BMS, in others they are given as complementary foods during weaning. Improper food selection at this stage is associated with a high prevalence of malnutrition in children under 5 years. Here we listed the traditional foods from four continents and compared them with human milk based on their dietary contents. Vitamins such as thiamine (~[2–10] folds), riboflavin (~[4–10] folds) and ascorbic acid (<2 folds) contents of Asian and African foods were markedly lower. In order to extend the search for foods that includes similar dietary constituents as human milk, we designed a strategy of screening 8654 foods. 12 foods were identified and these foods were evaluated for their ability to meet the daily nutritional requirement of breastfed and non-breastfed infants during their first year of life. Genome-scale models of infant’s hepatocytes, adipocytes and myocytes were then used to simulate *in vitro* growth of tissues when subjected to these foods. Key findings were that pork ham cured, fish pudding, and egg lean white induced better tissue growth, and quark with fruit, cheese quarg 45% and cheese cream 60% had similar lactose content as human milk.

## Introduction

Human breast milk is ideal for supporting growth and development of infants^1–3^. The World Health Organization (WHO) recommends mothers to exclusively breastfeed their child during the first 6 months of life^4, 5^. Several sociodemographic, biomedical, environmental and psychosocial factors contribute to the early cessation of breastfeeding^6, 7^. One such factor is early introduction of complementary foods^8, 9^. In some communities traditional foods and infant formulas are given as substitues^10^. Energy intake of some traditional foods are well below infant’s energy requirements^11, 12^. Unlike infant formula which is standardized with a small range of constituents^13^, human milk has a wide range of nutritional and non-nutritional constituents such as bioactive factors^14^. The choice of breast-milk substitutes (BMS)^15^ is critical and have faced several challenges including risk of infections^16^ and increased formula marketing^17^.

Complementary feeding is defined as the process starting, when breast milk alone is not sufficient to meet with the nutritional requirements of infants. Complementary foods are generally given between 6 to 24 months of age along with the breast milk^18^. The choice of complementary foods and feeding practices in developing or underdeveloped countries have limited scientific guidelines^19–24^. These guidelines must consider a number of issues such as time of introduction^25, 26^, types, order, amounts of foods given, and providing essential micro- and macromolecular contents^27^. Improper food selection is associated with a high prevalence of malnutrition in children under 5 years^28, 29^. Gathering all these facts together, selection of complementary foods with optimal nutritional values is critical^18^. Scientific food recommendations must be cost-effective, affordable, locally available and practical for low income populations, many of which are susceptible to malnutrition and obesity^30^.

Systems biology together with bioinformatics and food metabolomics has begun to emerge as essential tools in food science and nutritional research^31–33^. Mathematical models were designed to understand the critical constraints of nutritional recommendation^34–38^ and food intake pattern^39^. The models were used to evaluate the optimal nutrient density and thereby nutrient-adequate diets^39^ were proposed. In this context Genome-scale models (GEMs) are efficient tools for prediction of growth phenotypes in living cells exposed to different nutrients^40, 41^. Recently, Bordbar *et al*., used an integrative approach to model the multi-tissue interactions in human metbolism^42^. Moreover, integration and analysis of various high throughput datasets together with cutting-edge technologies have unveiled dietary biomarkers and elucidated their physiological role^43–45^.

In this study, we took an integrated computational approach to estimate the nutritional similarity and differences of the continent-wise frequently consumed or traditional foods with human milk based on their dietary contents and evaluated if these foods could be given as BMS to the infants. Food metabolomics^43^ data were collected and standardize to determine the chemical composition of these foods.

A food screening strategy was designed that identified various foods deemed optimal for satisfying the daily nutritional requirements of infants when given solely (BMS) or together with breast milk (complementary foods) during the first year of life. The selected foods and nutrients intake were also used to predict tissue growth using GEM simulations^42, 46–48^.

## Results

### Nutritional contents of traditional foods as compared to human milk

**Figure 1:**
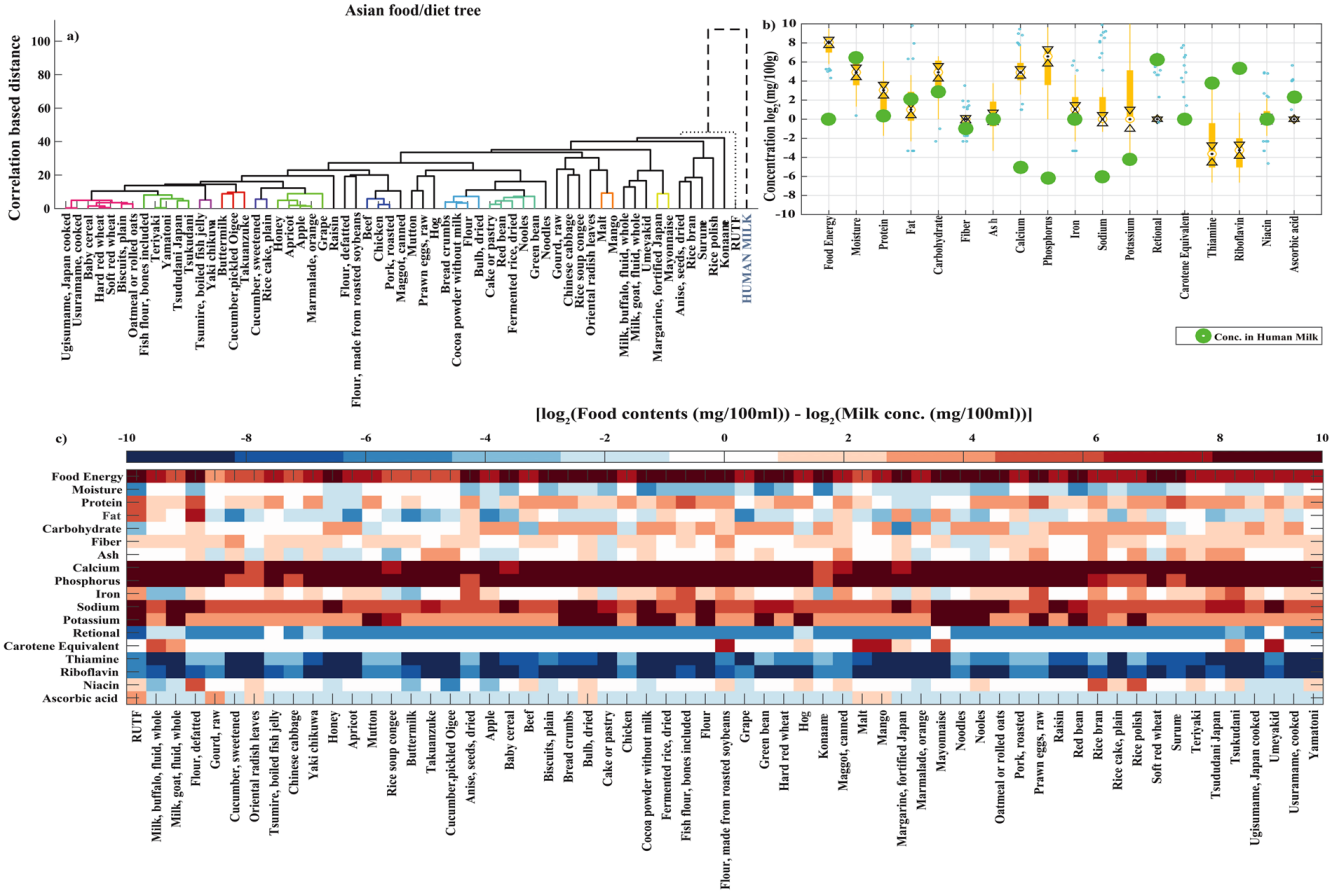
(**a**) Food tree of common and traditional Asian foods/diets. Each branch represent CBDM (γ) scaled between [0–100]. Cluster of similar foods with δ < 10 are color coded. Human milk or standard RUTF is represented as dotted lines. (**b**) Boxplot showing log of total dietary contents expressed in mg/100 g of foods. Black dot represents the median and cyan dots are the outliers. The concentration of the constituents available in human milk is marked with green dots.

The contents of the commonly consumed Asian foods showed distinct differences from human milk which is set as a reference (Fig. 1 panel a). The energy (~[4–10] folds), protein (~[2–6] folds) and carbohydrate (<4 folds) contents of these foods were found to be higher than in human milk; the fat and moisture contents were found to be similar. Higher content of minerals such as calcium (~[8–10] folds), phosphorus (~[8–10] folds), sodium (~[6–10] folds) and potassium (~[4–10] folds) were also observed. The vitamin contents such as thiamine (~[2–10] folds), riboflavin (~[4–10] folds) and ascorbic acid (<2 folds) were, however, markedly lower with a small difference in niacin (Fig. 1 panel b and panel c).

**Figure 2:**
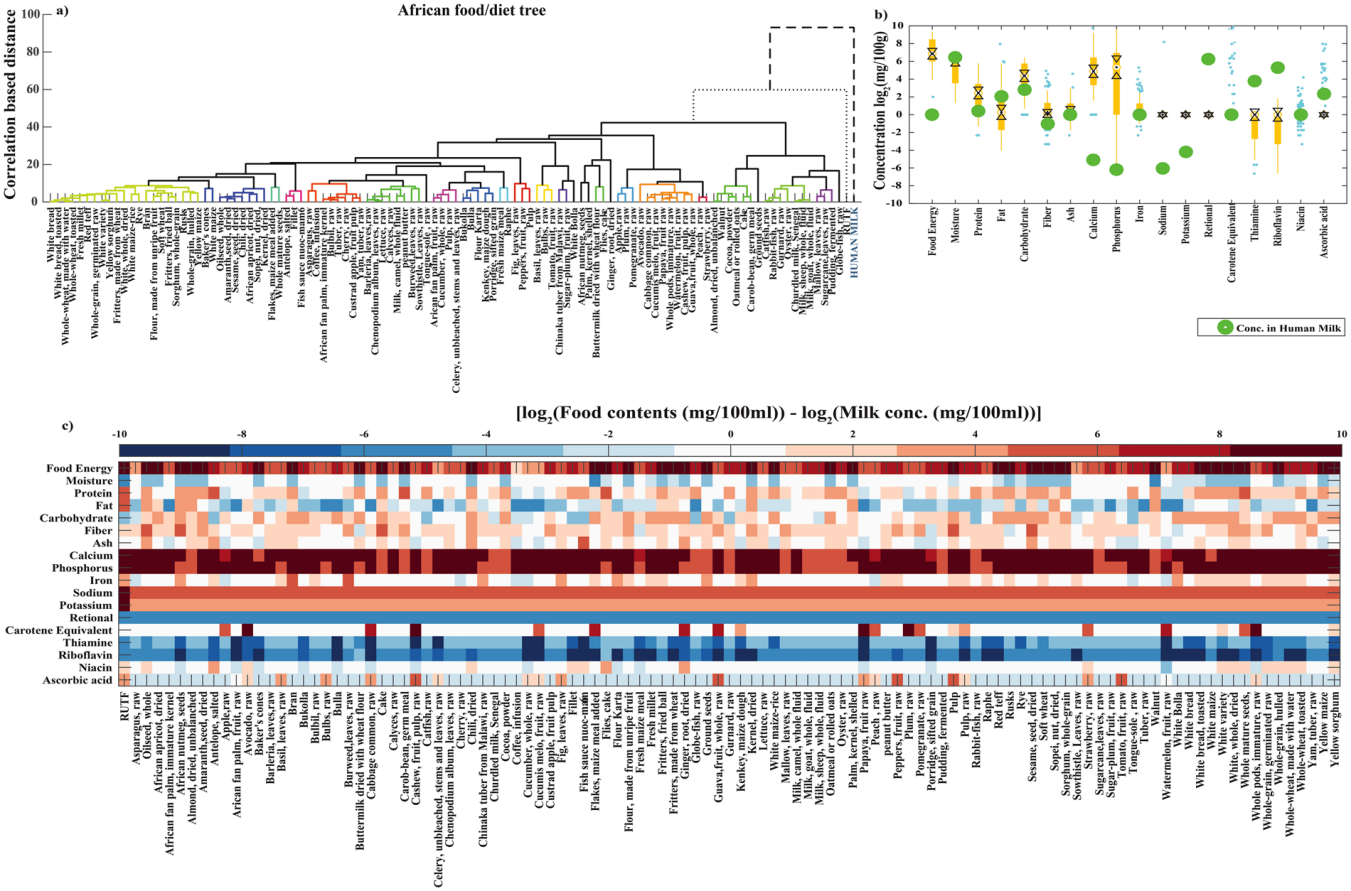
(**a**) Food tree of common and traditional African foods/diets. Each branch represent CBDM (γ) scaled between [0–100]. Cluster of similar foods with δ < 10 are color coded. Human milk or standard RUTF is represented as dotted lines. (**b**) Boxplot showing log of total dietary contents expressed in mg/100 g of foods. Black dot represents the median and cyan dots are the outliers. The concentration of the constituents available in human milk is marked with green dots.

Similarly, neither of the selected traditional African foods showed similarity with human milk (Fig. 2 panel a), and a similar pattern was found for traditional Asian foods (Fig. 2 panel b).

The contents of selected traditional American and European foods were also found to be dissimilar to human milk (Supplementary Figs 1 and 2). However, the ascorbic acid content was markedly higher than in Asian and African foods and more similar to that in human milk. Higher content of retinal and lower content of thiamine and riboflavin were also observed.

Apart from the traditional foods, we have also compared the contents of standard Ready-to-Use Therapeutic foods (RUTFs)^49^ with human milk. RUTFs are therapeutic foods given particularly as dietary supplements to children with severe acute malnutrition (SAM) or elderly persons with dietary insufficiency^49^. Lack of similarity was marked between contents of human milk and prescribed RUTFs. The proteins, fats, energy and minerals contents such as sodium, calcium, potassium, iron and phosphorous were markedly higher, whereas lower levels of carbohydrates and vitamins such as thiamine and riboflavin were found. Thus, sole administration of RUTFs as supplements might not be sufficient to fulfill the daily nutritional requirements of the infants. They are low in carbohydrates and vitamins that fuel the metabolic processes that are necessary for growth and development.

### Characterization of foods similar to human milk

**Figure 3:**
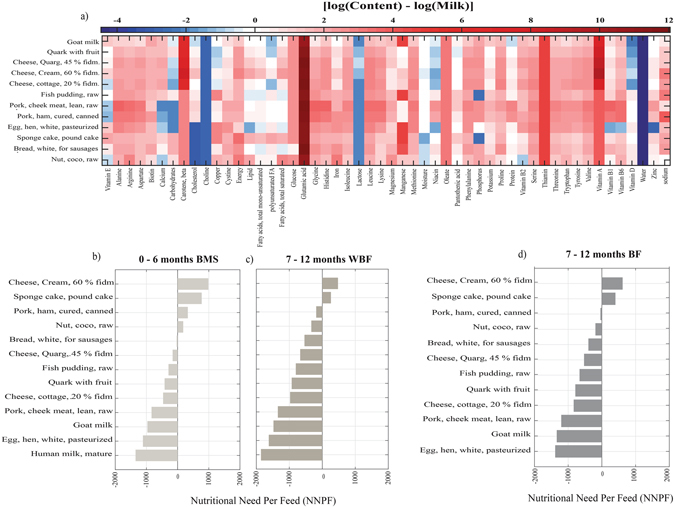
(**a**) Fold change in concentration of food constituents expressed in mg/100 g with respect to human milk. (**b**) Nutritional Need Per Feed (NNPF) scores of these foods when given to 0–6 months’ infants as breast milk substitutes (BMS). (**c**) NNPF scores of foods when given to 7–12 months infants as substitutes without breast feeding (WBF). (**d**) NNPF scores of foods when given to 7–12 months’ infants as complementary foods (with breast feeding, BF).

As neither of the traditional foods in any of these food groups showed high similarity in nutritional or dietary contents with human milk, we continued to search for complementary foods with high nutritional values. A computational approach was adapted for food screening (*see Methods*). Foods that showed at least 70% (δ > 0.70) correlation with human milk contents were selected. These foods were reviewed based on expert’s knowledge (team of clinicians and nutritionist) and infant’s capability to ingest these in any forms. 12 foods were selected because they met these criteria. The content and composition of these foods relative to human milk are shown in (Fig. 3 panel a and Supplementary Figs 3 and 4).

Most of these foods were dairy products, including goat milk (δ = 0.70125), quark with fruit (δ = 0.76115), and cheese (with quarg or creamed, δ ~ 0.74). Others include fish pudding 131 (δ = 0.745), pork cheek lean meat raw (δ = 0.7315), egg hen white (δ = 0.71847) and bread white sausages (δ = 0.72154) (Supplementary information).

### Capability of selected foods to cope with the infant’s daily nutritional requirements

The selected foods were evaluated to determine if they could fulfill the daily nutritional requirements of breastfed and non-breastfed infants during the first year of life.

Some of these foods such as cheese cream 60%, sponge cake, pork ham cured, and nut coco raw had positive Nutritional Need Per Feed (NNPF) scores when given as BMS to the infants (Fig. 3 panel b). Among these cheese cream 60%, sponge cake, pork ham cured were rated higher. The energy and most of the nutrients contents of these foods were well above the threshold of daily nutritional requirements of the infants when given at least once per day. Other foods such as cheese (quark and cottage 30%), fish pudding, and egg lean white and human milk showed negative NNPF values (Fig. 3 panel b). Most of the nutrients contained in these foods are therefore below the daily nutritional requirements and would have to be provided more frequently to meet with the nutritional requirements for infant’s growth. Some of these foods such as quark with fruit, cheese quarg 45% and cheese cream 60% showed similar lactose content as human milk (Fig. 3 panel a).

Cheese cream 60% and sponge cake had positive NNPF scores when given together with human milk as complementary foods to 7–12 months infants (Fig. 3 panel c). Moreover, these foods also had positive NNPF scores when given as substitutes to the non-breastfed infants of the same age (Fig. 3 panel d).

### Impact of selected foods on tissue growth

**Figure 4:**
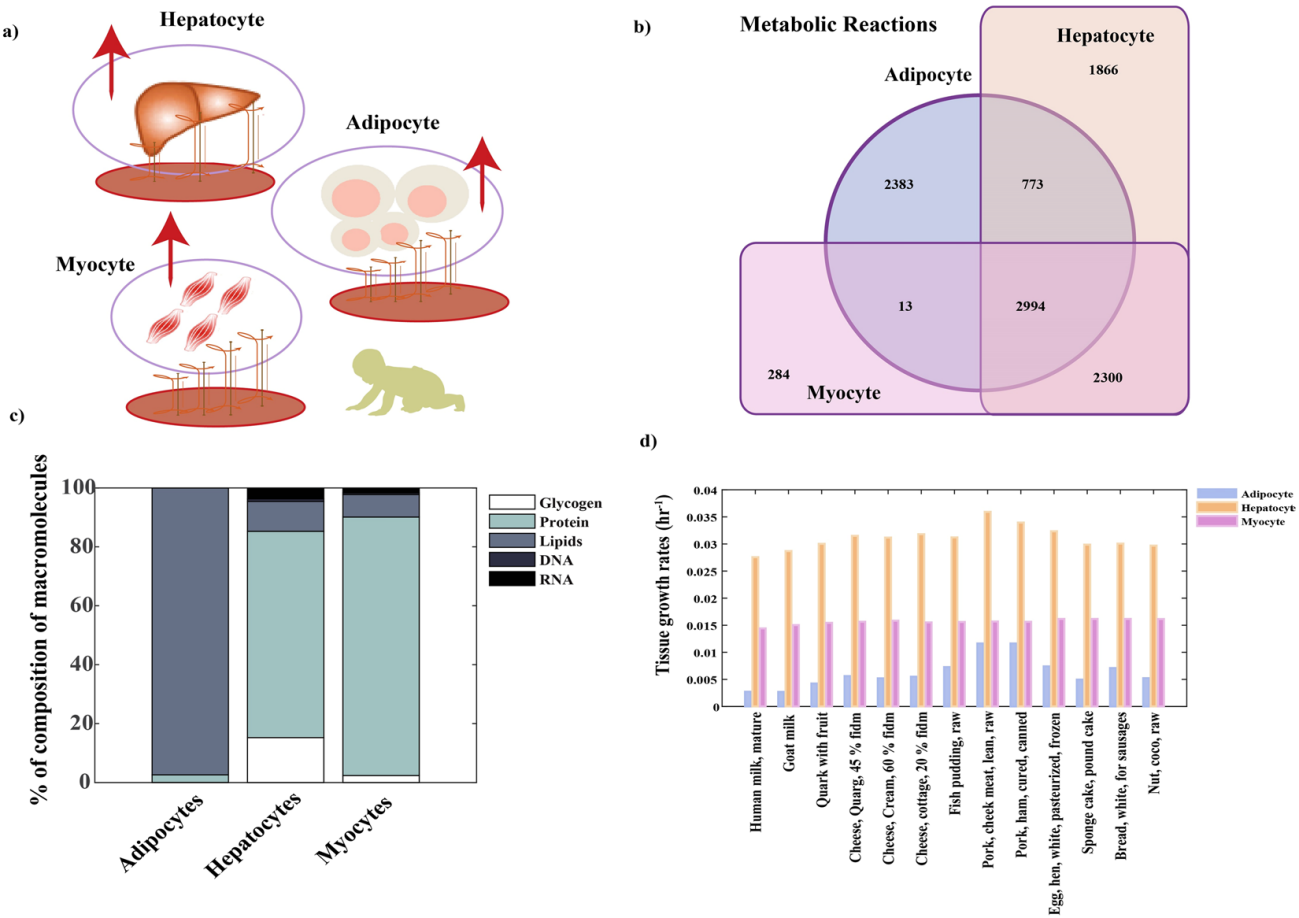
(**a**) Representation of different tissue models (GEMs) included in the analysis. (**b**) Similar or different metabolic reactions contained in the models. (**c**) Percentage of tissue composition and its macro-molecular contents (**d**) Growth rates (hr^−1^) of hepatocytes, myocytes and adipocytes.

Hepatocytes, adipocytes and myocytes are among the major tissues that orchestra human metabolic processes. The metabolic reactions contained in these tissues have been captured in genome-scale metabolic models (GEMs)^46–48^. The number of reactions and overlap between the three cell types along with their composition is summarized in Fig. 4 (panel b, panel c). The nutritional and metabolic demands of these tissues might be different in growing infants. These demands are fulfilled by the foods and essential nutrients which facilitates the growth and development. Thus, the macro- and micro molecular contents including essential nutrients present in these foods could be critical for growth and maintenance.

In order to estimate the growth of tissues with food intake and amount of nutrient content, a GEM modeling approach was adapted. The selected foods and their nutritional contents were used as dietary constraints and growth rates of specific tissues were estimated (*see Methods*). The predicted growth rates were compared with the *in vitro* maximum growth rates of hepatocytes^42^, adipocytes^42^ and myocytes^42^ measured experimentally. Higher growth rates of hepatocytes than myocytes and adipocytes with any these foods was observed. Pork ham cured, fish pudding, and egg lean white showed better growth of hepatocytes, adipocytes than human milk and other dairy products, whereas nominal growth differences in myocytes were observed.

## Discussion

WHO suggests that infants should be exclusively breastfeed until 6 months after birth. Several sociodemographic, biomedical, environmental and psychosocial factors contribute to cessation of breastfeeding. In some communities traditional family foods are introduced as alternatives to breastfeeding, in others they are given as complementary foods during weaning. Nutritionally inadequate infant foods and limited scientific evidence, education and dietary recommendations could lead to impaired growth, development and severe health-related disorders in infants. Moreover, dietary content and nutritional values of complementary foods have to be revised with the advent of new cutting-edge technologies and food metabolomics.

With several nutritional benefits, the nutritional content of human milk could serve as baseline for selection and screening of complementary foods. We have listed traditional and frequently consumed infant foods across four continents and compared them with human milk based on their dietary contents. We also estimated the nutritional similarity of these foods with human milk. Lack of similarity in dietary contents were marked between human milk and traditional foods in any of the food groups. The foods of Asian and African countries mostly include cereals such as maize, sorghum, millets and rice that contribute 40–60% of the total dietary energy (http://www.fao.org/). These foods contained higher carbohydrate, protein and energy contents as compared to human milk. However, vitamins such as thiamine, riboflavin and ascorbic acid content were markedly lower. Vitamins are essential for growth and nutrition^50–52^, and severe deficiency of thiamine and riboflavin had shown to reduce infant’s growth^51–53^. Therefore, use of some traditional or regular Asian and Africa infant foods could contribute to nutritional deficiencies, if they are used to replace breastfeeding.

As dietary contents of traditional foods were found to be dissimilar to human milk in any of the food groups, a search for new foods was conducted. About 8654 foods along with their metabolic profiles were screened against human milk, the foods that showed (δ > 0.70) were listed. Among different listed foods, quark or *‘Tvorog’* a firmer variety of quark found in Russia, Ukraine and Belarus has been recommended earlier for growing infants (http://www.rg.ru/) and cottage cheese is given as a complementary food. The selected foods include dairy products such as goat milk, quark with fruit, cheese (with quarg or creamed). Other included high protein diets such as fish pudding, pork cheek lean meat raw, egg hen white and bread white sausages which contained higher level of minerals (except calcium) and vitamins with moderate amount of carbohydrates. Neither of these foods leveled the lactose content of human milk (Fig. 3 panel a). We recommend a mixed diet regime of foods with high protein content together with cheese cream 60% or cheese quarg 45% to level the carbohydrate and lactose content of human milk. Some of these foods such as pork cheek lean meat raw, egg hen white and bread white sausages should, however, be fortified with calcium which is low in these foods compared with breast milk.

Excess or lack of nutrients with poor feeding practices might trade-off between over- and under nutrition in growing infants^54^. The selected foods were evaluated for their ability to cope with the daily nutritional requirements of the infants given per meal (100 g of food). These foods were divided into two categories based on NNPF score. Cheese cream 60%, sponge cake, pork ham cured, and nut coco raw had positive NNPF scores when given as BMS to infants during the first six months, these foods might be sufficient to meet the daily nutritional requirements given at least once per day. Moreover, cheese cream 60%, sponge cake also had positive NNPF values when given as complementary foods to breastfed or substitutes to non-breastfed infants of 7–12 months. On the other hand cheese (quark and cottage 30%), fish pudding, and egg lean white and human milk showed negative NNPF values if given as BMS, it means that they are insufficient to cope with the daily nutritional needs when given one meal per day. The NNPF index could be extended to decide the frequency of feeding and thereby aid to formulate personalized diets.

Foods aids in growth and maintenance of the tissues, which in turn facilitate the growth of individuals. Hepatocytes, myocytes, adipocytes carry most of the metabolic processes in the human body, GEMs of these tissues^46–48^ were designed and deployed to estimate the maximum growth of these tissues subjected to selected foods and nutrients intake given per meal. The nutritional content of each of these foods were set as dietary constraints limited by uptake rates of the tissues (*see Methods)*. The predicted growth rates were compared with the *in vitro* maximum growth rates of hepatocytes^42^, adipocytes^42^ and myocytes^42^ measured experimentally. Higher growth rates were marked for hepatocytes than myocytes and adipocytes respectively, with any of these foods, which is consistent with the larger metabolic flexibility of these cells. Moreover, pork ham cured, fish pudding, and egg lean white showed higher tissue growth than human milk and other dietary products. Some nominal variations were found in growth of myocytes when subjected to these foods. This could be speculated as most of the protein (amino acids) content in these foods were well above the minimum intake requirements for growth of myocytes (Fig. 3 panel a).

As the choice of complementary foods are also guided by the local availability and cultural diversity, we referred to the contextual complementary feeding recommendations (CFRs)^55^ based on locally available foods in Indonesia and Asia Pacific region^56^. Among 12 identified foods we recommend goat milk, cheese (with quark, cream, cottage, and fruit), egg hen white and fish puddings for these populations. Along with these foods, bread white and cheek meats are suggested as complementary foods in South Africa^57^.

The study provide a computational approach for identification of food substitutes with nutritional value equaling a given food, e.g. breast milk, and can hereby contribute to the knowledge base for selection and evaluation of complementary foods based on their nutritional contents. The dietary regime of Asia and Africa should be revised and food with high nutritional values should be included to minimize the chance of malnutrition or related nutritional disorders. The proposed foods could aid in the formulation of complementary foods or substitutes with lack of breastfeeding under physician’s recommendation and supervision. However, the non-nutritional component (bioactive compounds) and immunological factors of these foods are still to be evaluated.

## Materials and Methods

### Selection of foods across continents

Food composition data (FCD) for Asia, Africa, America and Europe foods were obtained and standardized. Asian foods were selected from 14 different food groups according to local eating habits^58^ (http://www.fao.org/); at least one food was selected from each group. Such criteria was adapted for selection of African food^59^ (http://www.fao.org/). American food content was obtained from *Planetary Health*. *Inc*. *2011* (based on USDA National Nutrient Database (http://ndb.nal.usda.gov//) and similar criteria for food selection was adapted. To the best of our knowledge no standard European food composition table or integrated food datasets is available to date^60^. McCance and Widdowson’s reported composition of 1200 foods consumed in UK^61^. These foods were compared with other European foods reported in (http://www.eurofir.org/) and a consensus list was prepared. The listed foods were divided into various groups based on their contents. One or more food(s) from each group was selected (Supplementary Table 1). The average nutritional composition of human breast milk was obtained from United Nations University Centre (http://archive.unu.edu/unupress/food/8F174e/8F174E04.htm). An estimate of the nutritional composition of breastmilk, derived from extensive sampling of breast milk from women in Britain and Gambia.

The selected list of Asian foods comprised of rice products (flour, barn, and cake), wheat flour, roasted soy flour, that are often given as first choice of complementary foods in Asia Pacific region^56^; other foods included goat milk and buffalo milk. The selected traditional African foods list included porridge, puddings, maize flour, peanut butter, butter milk dried with wheat flour, and goat milk that were commonly given as complementary food in South Africa^57^. Beans, avocado, banana, mango, oat, barley were among the list of American foods given as complementary food in Central America (http://www.eatrightpro.org/resource/practice/practice-resources/international-nutrition-pilot-project/breast-feeding-and-complementary-nutrition). Similarly, selection of European complementary foods were guided by recommendation of European Society of Paediatric Gastroenterology, Hepatology and Nutrition (ESPGHAN) committee^23^.

### Classification of similar and dissimilar foods

Correlation-Based Distance Measure (CBDM) denoted by γ is a multivariate approach to estimate degree of similarity and dissimilarity among different foods/diets. CBDM is given by:

1 $${\rm{\gamma }}=1-{\rm{\delta }}\,$$

where δ is sample Spearman’s correlation between concentrations of food constituents and human milk, treated as sequences of values. CBDM was used to determine degree of closeness of food with respect to Human milk and Ready-to-use therapeutic foods (RUTFs)^49^.

Principal component analysis (PCA) was used as an alternate measure to validate clusters of similar foods suggested by CBDM. However, PCA could not estimate the degree of similarity and thus CBDM was considered for classification and further analysis.

### Selection of foods similar to human milk

FooDB.ca (http://foodb.ca/) is a comprehensive resource that provides information about food composition, micro and macronutrients. About 8654 food/diets with measured macro or micro molecular contents were extracted (Supplementary Table 2). Spearman’s correlation (δ) was used to estimate the correlation among the food constituents with respect to human milk (set as reference). Foods with δ > 0.70 were scrutinized based on expert’s (dietician and clinician) knowledge and published guidelines, if they could be given to the infants in any form.

### Nutritional Need Per Feed (NNPF)

The daily nutritional requirement of infants was obtained and tabulated from USDA (https://wicworks.fns.usda.gov/wicworks/Topics/FG/CompleteIFG.pdf). A comprehensive plot of the daily nutritional requirements of the 0–6 months and 7–12 months healthy infants are shown in (Supplementary Fig. 5a and b) respectively. Nutritional Need Per Feed (NNPF), of a particular food intake was estimated by:

2 $$NNPF=\sum _{i=1}^{N}{[A1]}_{i}-{[A2]}_{i}$$

where [A1] is the concentration of ‘i^th^’ nutrient in 100 g of food and [A2] is the daily nutritional requirements estimated by USDA. Negative and positive NNPF values determines deficiency or surplus of nutrients available from the foods given per feed to meet with the daily nutrients requirements of the infants. Thus, NNPF is an index that indirectly determines the over or under representation of nutrients contained in the food to satisfy daily demand for growth and development.

### Genome-scale models and tissue growth

The growth of a tissue is a function of its cellular content or biomass, which comprises of macro and micro-molecules^42^. The tissue growth rate is given by:

3 $$\mathrm{dg}/\mathrm{dt}=(\sum _{i=1}^{n}a{X}_{{\rm{i}}}+\sum _{i=1}^{n}b{Y}_{{\rm{i}}}+\ldots +\sum _{i=1}^{n}k{K}_{{\rm{i}}})/\mathrm{dt}$$

where dg/dt is the growth rate (*day* ^−*1*^ *or hr* ^−*1*^ *or min* ^−*1*^ *or sec* ^−*1*^); a, b, k, denotes *mmol* of macro molecules (*eg*. glycogen, protein, fats, DNA, RNA etc.) per gram dry weight (*gDW*) of the tissue. X, Y, K denotes i^th^ micro molecules (amino acids. TAG, lipids, cAMP, TMP, *etc*.); that are either degradation products or monomers of macromolecules present in the cell.

GEMs of hepatocytes^47^ (Supplementary Dataset 1), myocytes^48^ (Supplementary Dataset 2), adipocytes^46^ (Supplementary Dataset 3) were obtained and growth equation was formulated. Estimation of growth coefficients (gc) were derived^41, 42^ using tissue composition data available^42, 62, 63^. Derivation of gc including the macro and micro-molecular contents is detailed in the (Supplementary Figs 6–8).

Growth rate (hr^−1^) of each tissue subjected to different foods was estimated by Flux Balance Analysis (FBA)^64^; with an assumption that the maximum nutrient utilization was bounded by concentration of the nutrient available in the foods. At steady state, the rates of reactions contributing to the growth, also known as fluxes (v) and stoichiometry [S] of all the metabolites involved in these reaction tends to zero. The growth was set as an objective function which was maximized.

4 $${\rm{\max }}({C}^{T}\cdot v)$$

5 $$subject\,to\,S.v=0$$

6 $$lb < v < ub$$

where C^T^ is weight vector, lb and ub are upper and lower bounds of metabolite utilization by each tissue respectively. A tutorial and primer for linear programming and FBA is available in ref. 64. The tissue growth simulations were performed with RAVEN Toolbox^65^. The MATLAB code sets and its usage deployed on the datasets and GEMs are made available on request.

## Electronic supplementary material

Supplementary information

Supplementary Table.1

Supplementary Table.2

Supplementary Dataset.1

Supplementary Dataset.2

Supplementary Dataset.3
