# Supplementary material for: Selection of complementary foods based on optimal nutritional values
Source: Sci Rep. 2017 Jul 14;7:5413. doi: 10.1038/s41598-017-05650-0 (PMC5511280; doi:10.1038/s41598-017-05650-0)
Supplement: Supplementary file 1 — Supplementary information [file 41598_2017_5650_MOESM1_ESM.pdf]

Selection of complementary foods based on optimal nutritional values

Partho Sen[1], Adil Mardinogulu[1,2], Jens Nielsen [1,3*]

[1]Department of Biology and Biological Engineering, Chalmers University of Technology. Kemivägen 10, SE-412 96, Göteborg, Sweden.

[2] Science for Life Laboratory, KTH - Royal Institute of Technology, SE-171 21, Stockholm, Sweden

[3]Novo Nordisk Foundation Center for Biosustainability, Technical University of Denmark, DK2800 Lyngby, Denmark

[*]Corresponding author: Jens Nielsen

Email: nielsenj@chalmers.se

Supplementary information

1.a) Similarity or dissimilarity of American foods with human milk

Most of these foods were rich in phosphorous, sodium, potassium and calcium. They also contained vitamin A, B1, B2 and C (Fig.1. panel b). But again neither of this foods were similar with human milk or RUTF (Fig.1. panel a).

b) Similarity or dissimilarity of European foods with human milk

Standardization of European food based on mineral and vitamin content is complex. Therefore, different foods were compared based on macro-molecular content. The carbohydrate content of these foods/diets were remarkably low. High fat content was marked when compared with other food groups (Fig.2. panel b).

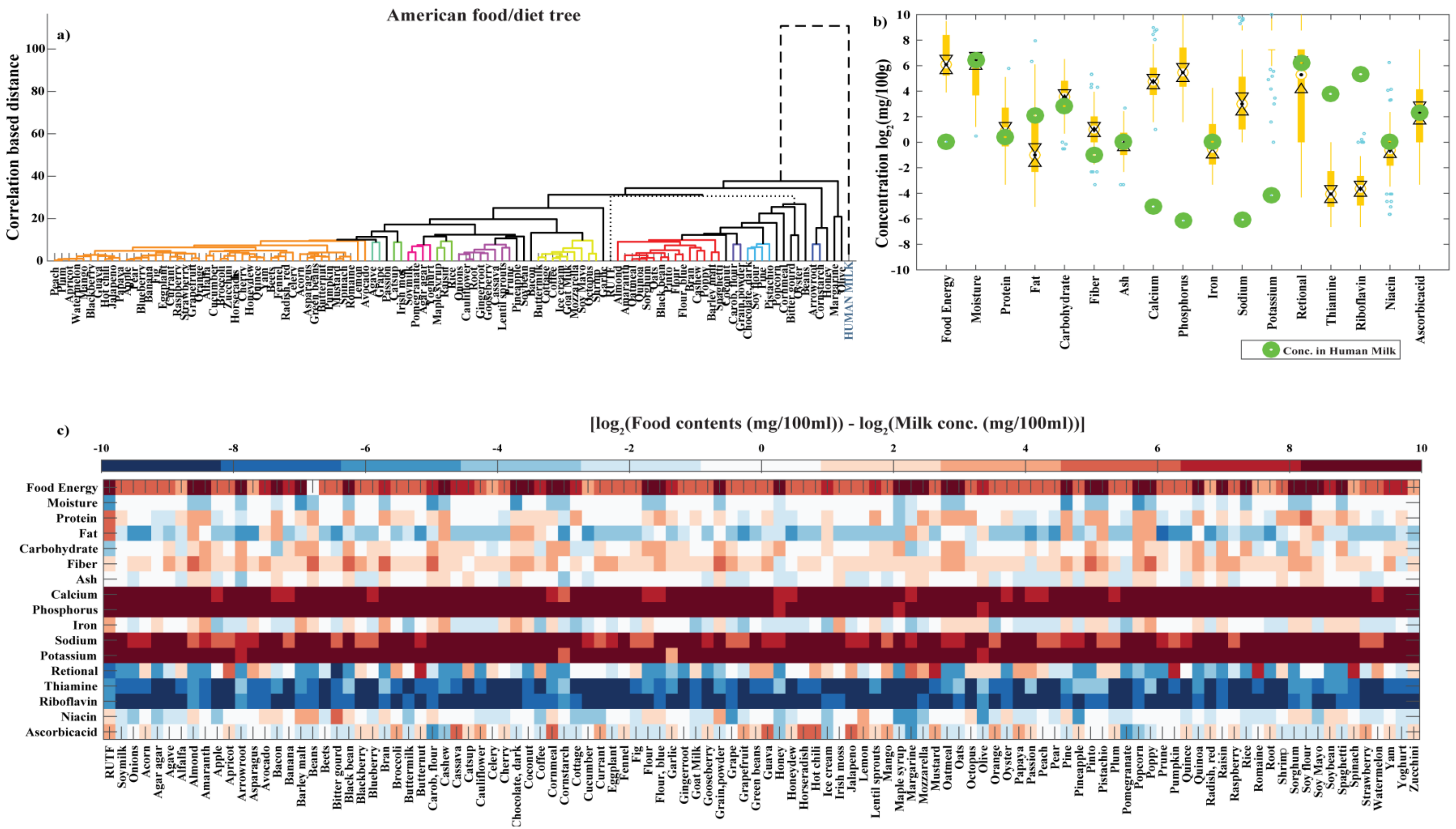

Fig.1 a) Food tree of common and traditional American foods/diets. Each branch represent CBDM (γ) scaled between [0-100]. Cluster of similar foods with δ < 10 are color coded. Human milk or standard RUTF is represented as dotted lines. b) Boxplot showing log of total dietary contents expressed in mg/100g of foods. Black dot represents the median and cyan dots are the outliers. The concentration of the constituents available in human milk is marked with green dots.

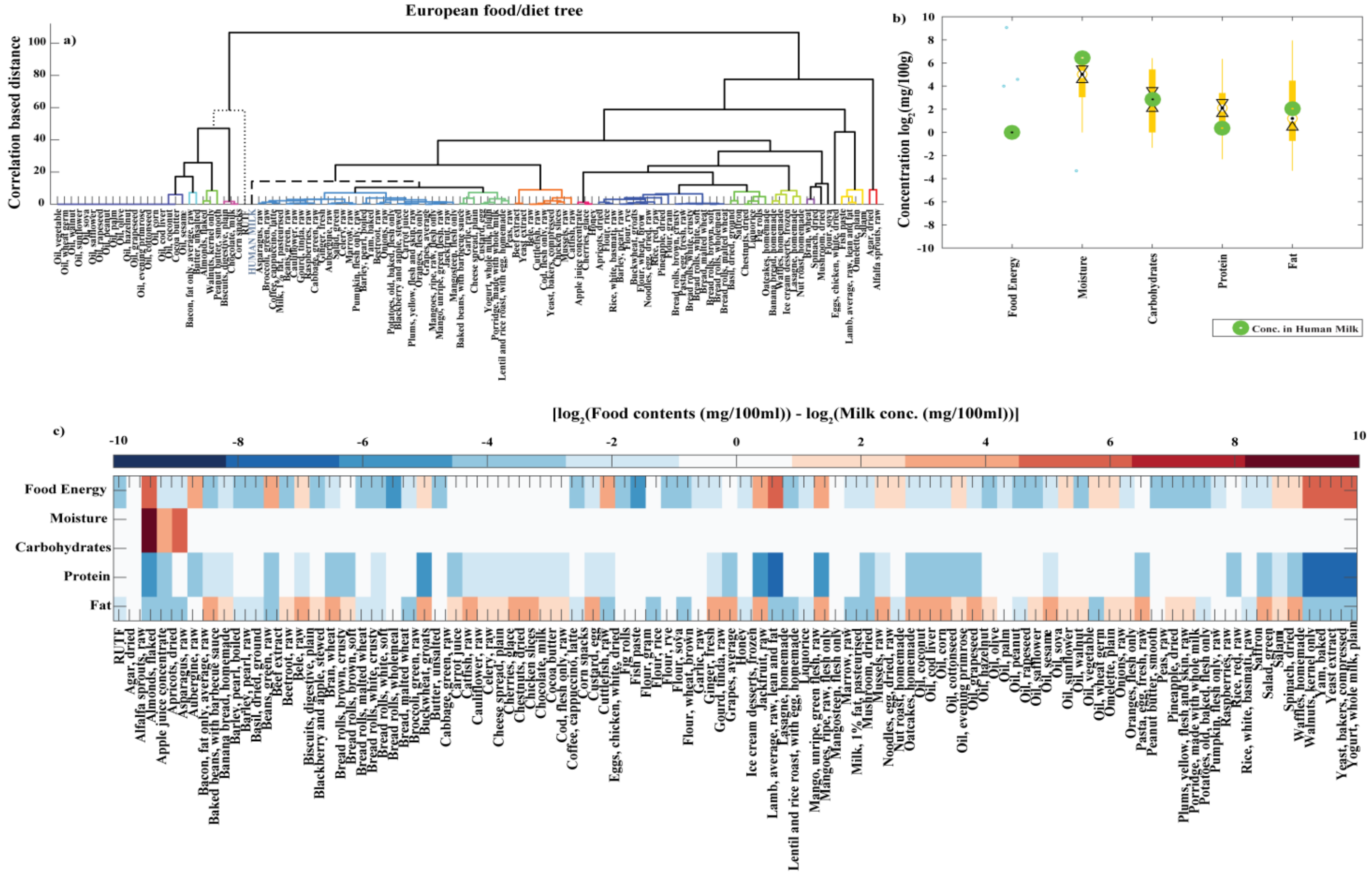

Fig.2 a) Food tree of common and traditional European foods/diets. Each branch represent CBDM (γ) scaled between [0-100]. Cluster of similar foods with δ < 10 are color coded. Human milk or standard RUTF is represented as dotted lines. b) Boxplot showing log of total dietary contents expressed in mg/100g of foods. Black dot represents the median and cyan dots are the outliers. The concentration of the constituents available in human milk is marked with green dots

2. Selected food and their dietary contents or metabolite levels relative to human milk

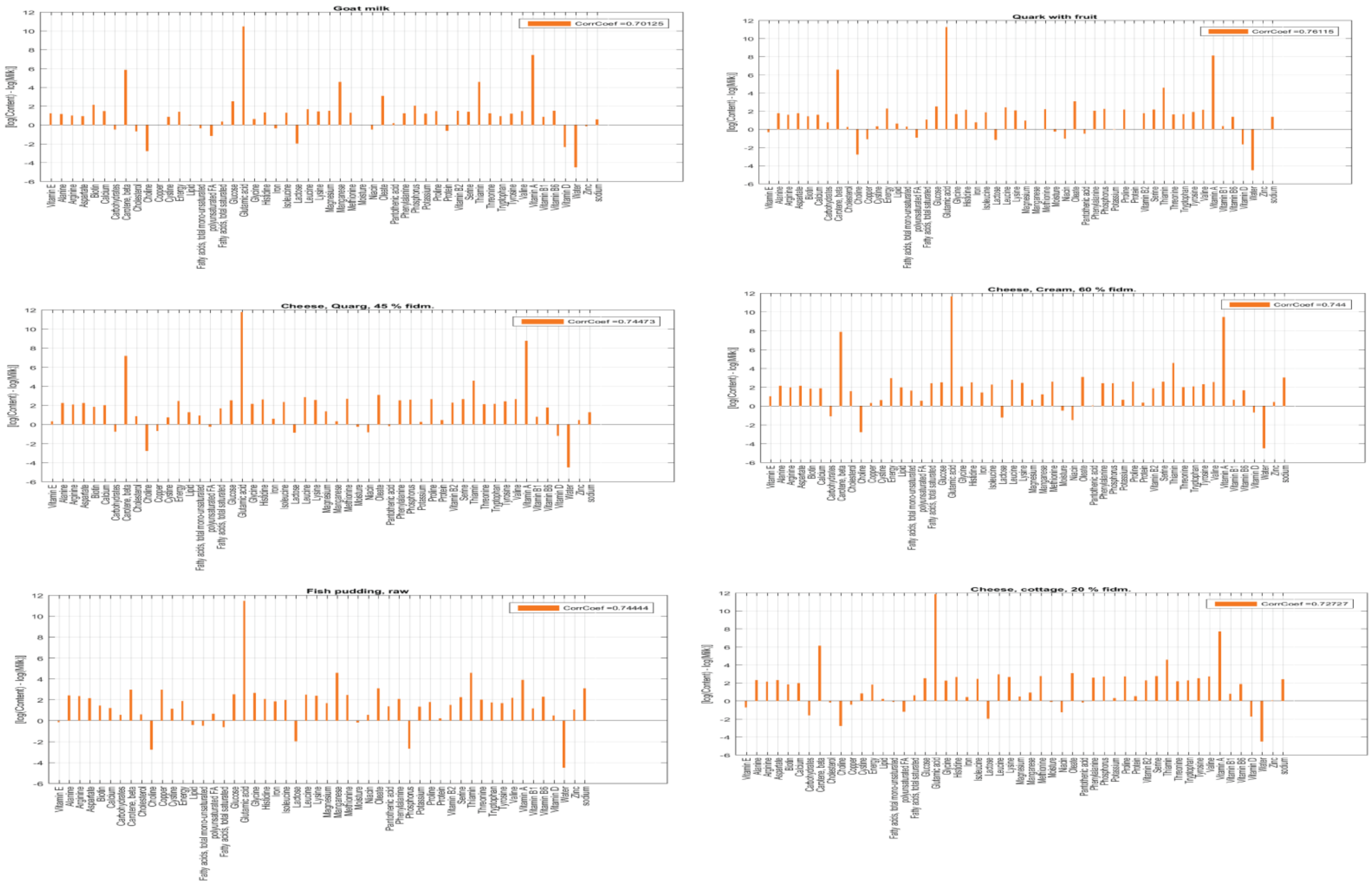

Fig.3. Bar plots of six selected foods with 70-76 % of correlation with human milk. Each bar represents relative difference ([log(content in food) – log(content in milk)]) of the metabolite level in the food and human milk.

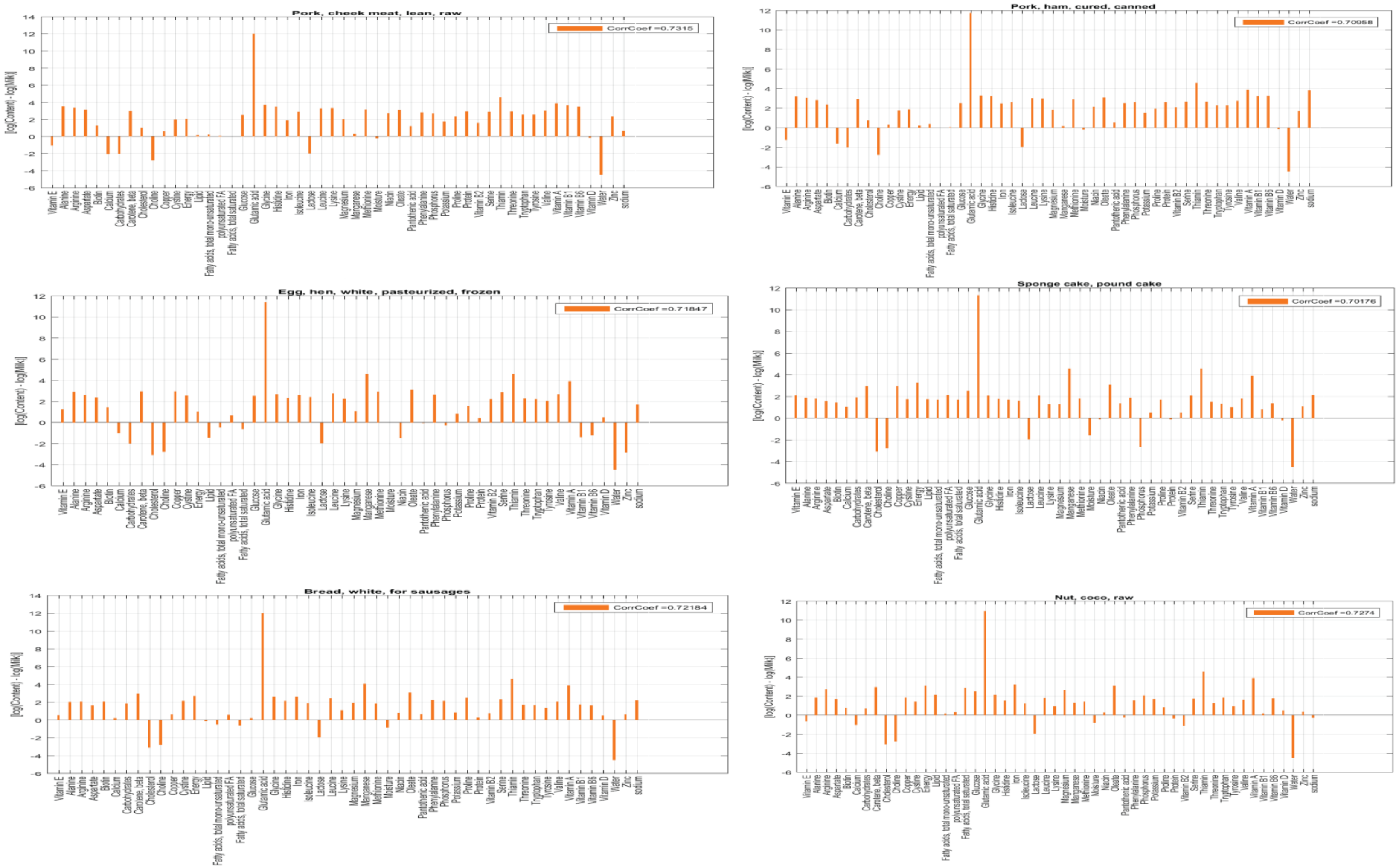

Fig.4. Bar plots of six selected foods with 70-73 % of correlation with human milk. Each bar represents relative difference ([log(content in food) – log(content in milk)]) of the metabolite level in the food and human milk.

3. Infants nutritional demands and feeding

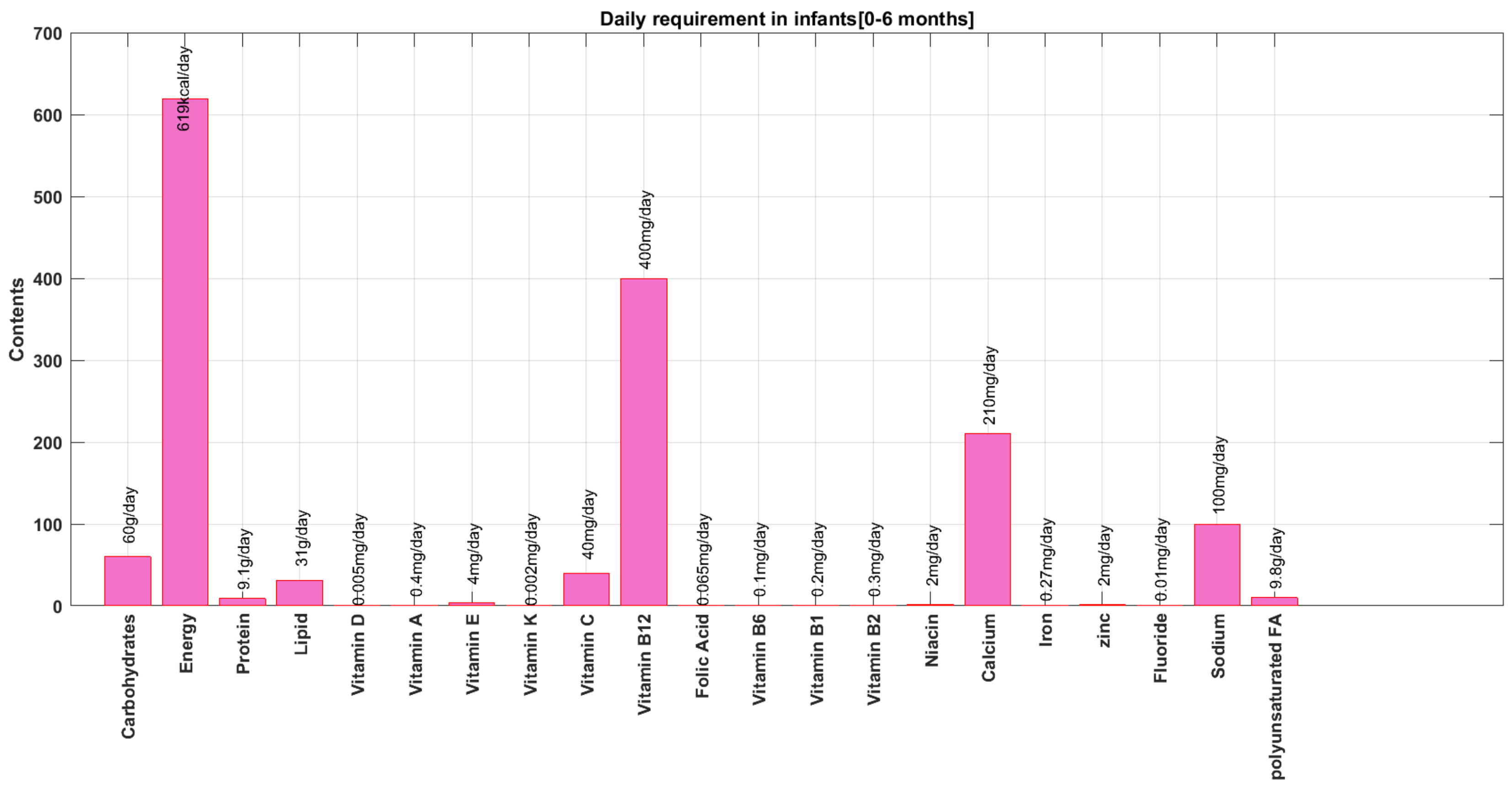

Fig.5a. Daily nutritional requirements of healthy infants of age 0-6 months. Data source (*https://wicworks.fns.usda.gov/wicworks/Topics/FG/CompleteIFG.pdf*)

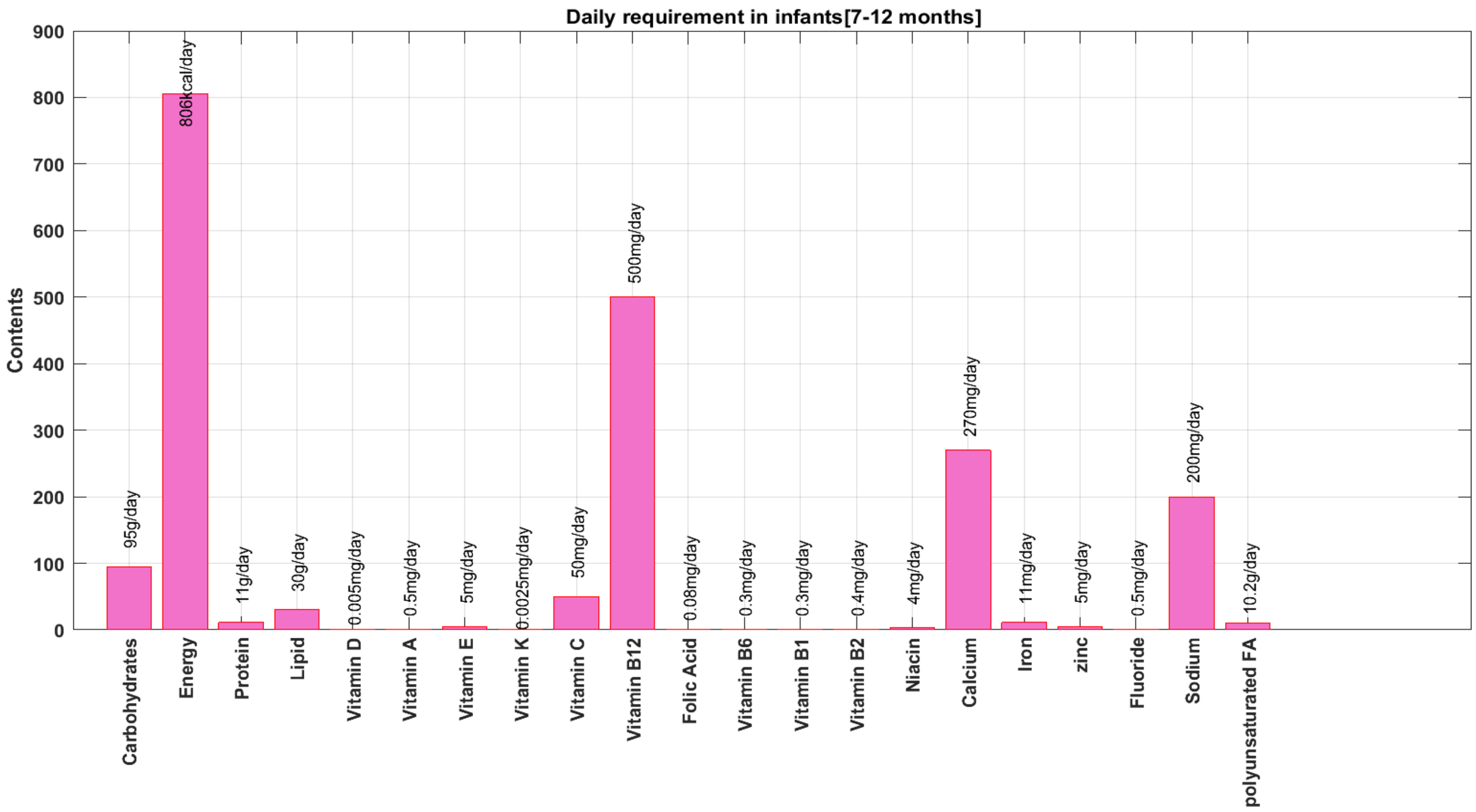

Fig.5b. Daily nutritional requirements of healthy infants of age 7-12 months. Data source (*https://wicworks.fns.usda.gov/wicworks/Topics/FG/CompleteIFG.pdf*)

4. Tissue composition and growth

As discussed in the main text, the growth of a tissue is a function of its cellular content or biomass, which comprises of macro and micro molecules[1].

4.a) Estimation of growth coefficients of Adipocyte (Fat cells)

The macromolecular composition of adipocyte was measured in several studies [2-5]. Biopsy of human adipocytes in normal, lean and obese subjects were obtained and analyzed for water, fat, protein, potassium and sodium contents[3]. Davidson *et al.*,[6] stated that, the number of fat storing cells are strictly limited; with the increase of fat contents the adipocytes were distended. Other studies contradicted with the hypothesis, therefore showed the significance of increase in number of fat cells with the increase of cellular contents[3]. In case of lack of experimental data, this stands as an approximation for estimation of adipocyte content/biomass with different body weights. Thomas *et.al* [3], established the relationship between weight of adipocytes and macromolecular contents[3] ; there was an increase in fat content of adipocytes with the increase in body weight. *Bodbar et al.*, [1] obtained the total macro and micro molecular contents of adipocyte and estimated the chemical composition.

To the best of our knowledge, no experimental data about the chemical composition of infant's adipocyte at micro molecular level are available. Only fatty acid profiling of adipocytes were performed in Spanish infants[7].Accumulating all the findings, data and body composition of the infants[8-10], we derived the adipocyte biomass and thereby formulated the growth equation (eq.1). Estimation of biomass, chemical composition formulae were obtained from Forster *et al.*,[11]. The g/g % of the compounds were converted to mmol/gDW of Tissue normalized for water contents. The growth of adipocytes is given by:

$G_{adipocyte}$ = 2.7037e-05 alanine + 1.2858e-05 arginine + 2.899e-05 asparagine + 2.8792e-05 aspartate + 5.3838e-06 cysteine + 3.5517e-05 glutamate + 3.5517e-05 glutamine + 4.1157e-05 glycine + 5.9162e-06 histidine + 1.3213e-05 isoleucine + 2.6209e-05 leucine + 1.7157e-05 lysine + 4.0033e-06 methionine + 1.201e-05 phenylalanine + 2.0549e-05 proline + 1.7078e-05 serine + 1.4731e-05 threonine + 1.2838e-05 tryptophan + 8.0264e-06 tyrosine + 1.8833e-05 valine + 6.382 PC-LD pool + 3.7241 PE-LD pool + 0.21052 PS-LD pool + 0.041929 lauric acid + 0.079666 myristic acid + 0.49057 palmitate + 0.081762 stearate + 0.27464 linolenate + 0.050005 DNA + 0.0039491 RNA + 4.0524 ATP => Biomass + 4.0524 ADP + 4.0524 H+ + 4.0524 Pi + 0.000891 PPi. eq.(1)

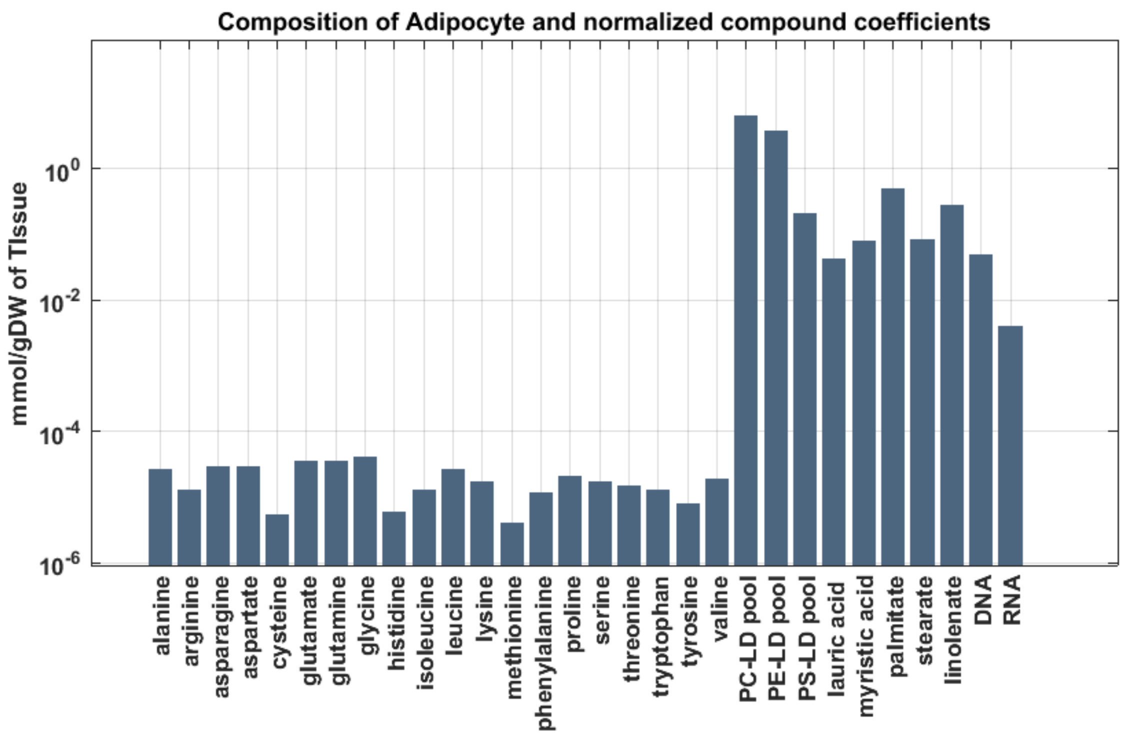

Fig.6. Bar plot of growth coefficients of adipocytes or the molecular content in mmol/gDW/Tissue normalized for water contents. PC-LD, PE-LD,PS-LD pool denotes phosphatidyl choline, phosphatidyl ethanolamine and phosphatidyl serine pools respectively.

4.b) Estimation of growth coefficients of Myocyte (Skeletal Muscle)

Different techniques were used to measure the chemical composition of the myocyte [12-17]. Bordbar *et al.*, estimated the biomass composition of human myocyte[1]. In a clinical trial, phospholipid profiles of skeletal muscle have been measured in Spanish infants at infancy and early childhood[7].

No experimental data about the chemical composition of infant's myocyte at micro molecular level are available till date. All these data and findings were used to estimate the growth coefficients of myocytes which is shown in Fig.7. The growth equation of myocyte is given by eq.(2).

$G_{myocyte}$= 0.5222 alanine + 0.25874 arginine + 0.00060172 asparagine + 0.65329 aspartate + 0.00017192 cysteine + 0.011218 glutamate + 0.84601 glutamine + 0.57864 glycine + 0.14699 histidine + 0.25573 isoleucine + 0.49904 leucine + 0.48288 lysine + 0.10758 methionine + 0.19362 phenylalanine + 0.31427 proline + 0.30185 serine + 0.32445 threonine + 4.298e-05 tryptophan + 0.1249 tyrosine + 0.3618 valine + 0.0011503 cholesterol + 0.00083433 PE-LD pool + 0.0014855 PC-LD pool + 0.00029914 PI pool + 0.00010936 PS-LD pool + 0.013037 myristic acid + 0.25807 palmitate + 0.2461 stearate + 0.25541 linolenate + 0.074703 DNA + 0.17173 RNA + 0.17523 glycogen + 8.9937 ATP => Biomass + 8.9937 ADP + 8.9937 H+ + 8.9937 Pi + 0.0705 PPi. eq.(2)

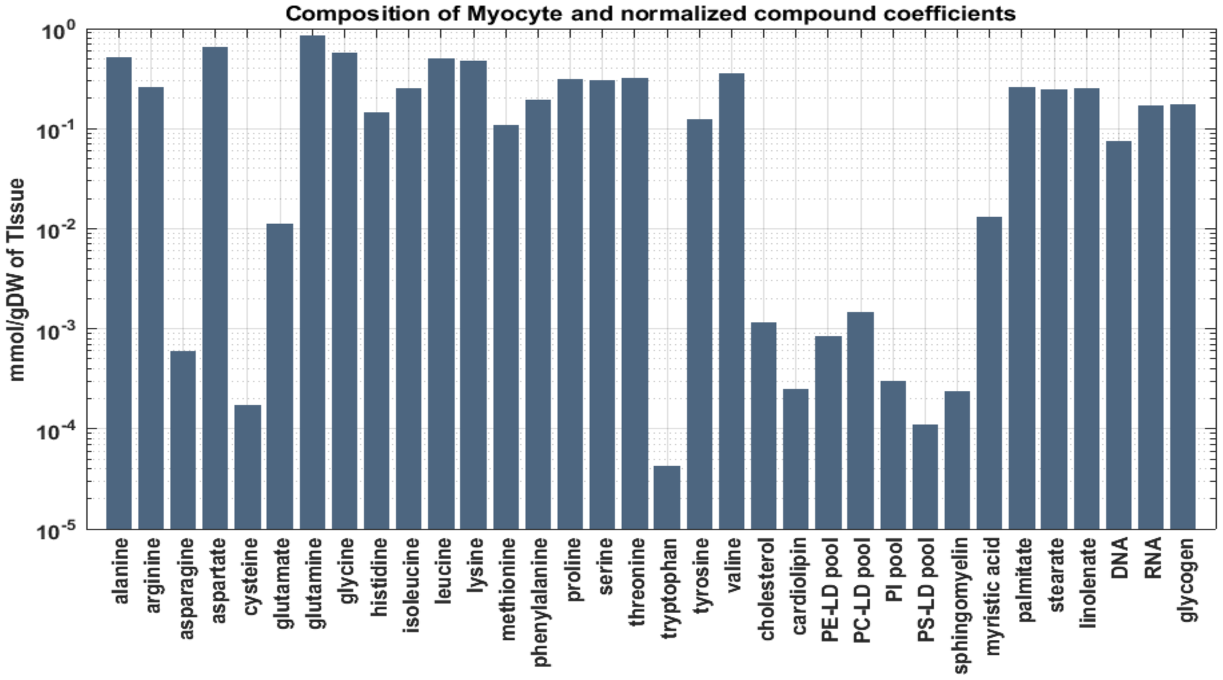

Fig.7. Bar plot of growth coefficients of myocyte or the molecular content in mmol/gDW/Tissue normalized for water contents. PC-LD, PE-LD,PS-LD pool denotes phosphatidyl choline, phosphatidyl ethanolamine and phosphatidyl serine pools respectively.

4.c) **Estimation of growth coefficients of Hepatocyte (Liver cells)**

To the best of our knowledge chemical composition of infant's liver has not been reported. Bordbar *et al.*, derived and estimated the biomass composition of human hepatocytes[1]. Chemical analysis of miniature pig liver model[18] also gives an opportunity to characterize the molecular contents as a proxy for infant's hepatocyte. The growth of the liver is proportional to age of the infants[19] irrespective of gender. Thus, weight of liver was extrapolated from the body weight and gender[20]. All these data were used to estimate the growth coefficients of hepatocyte. Growth of hepatocyte is given by eq.(3).

$G_{hepatocyte}$ = 0.0061568 alanine + 4.81e-05 arginine + 0.0006253 asparagine + 0.035931 aspartate + 6.253e-05 cysteine + 0.0087061 glutamate + 0.0099086 glutamine + 0.007215 glycine + 0.0014911 histidine + 0.0001924 isoleucine + 0.0005772 leucine + 0.000481 lysine + 9.62e-05 methionine + 0.0001924 phenylalanine + 0.00093314 proline + 0.001924 serine + 0.0010582 threonine + 4.81e-05 tryptophan + 0.0002886 tyrosine + 0.0006253 valine + 0.002686 lauric acid + 0.0068322 myristic acid + 0.010071 palmitate + 0.007187 stearate + 0.017842 linolenate + 0.06015 cholesterol + 0.025041 PI pool + 0.1394 PE-LD pool + 0.044751 PS-LD pool + 0.14045 PC-LD pool + 0.16887 glycogen + 0.5668 DNA + 1.6654 RNA + 26.4191 ATP => Biomass + 26.4191 ADP + 26.4191 H+ + 26.4191 Pi + 0.1431 PPi
eq.(3)

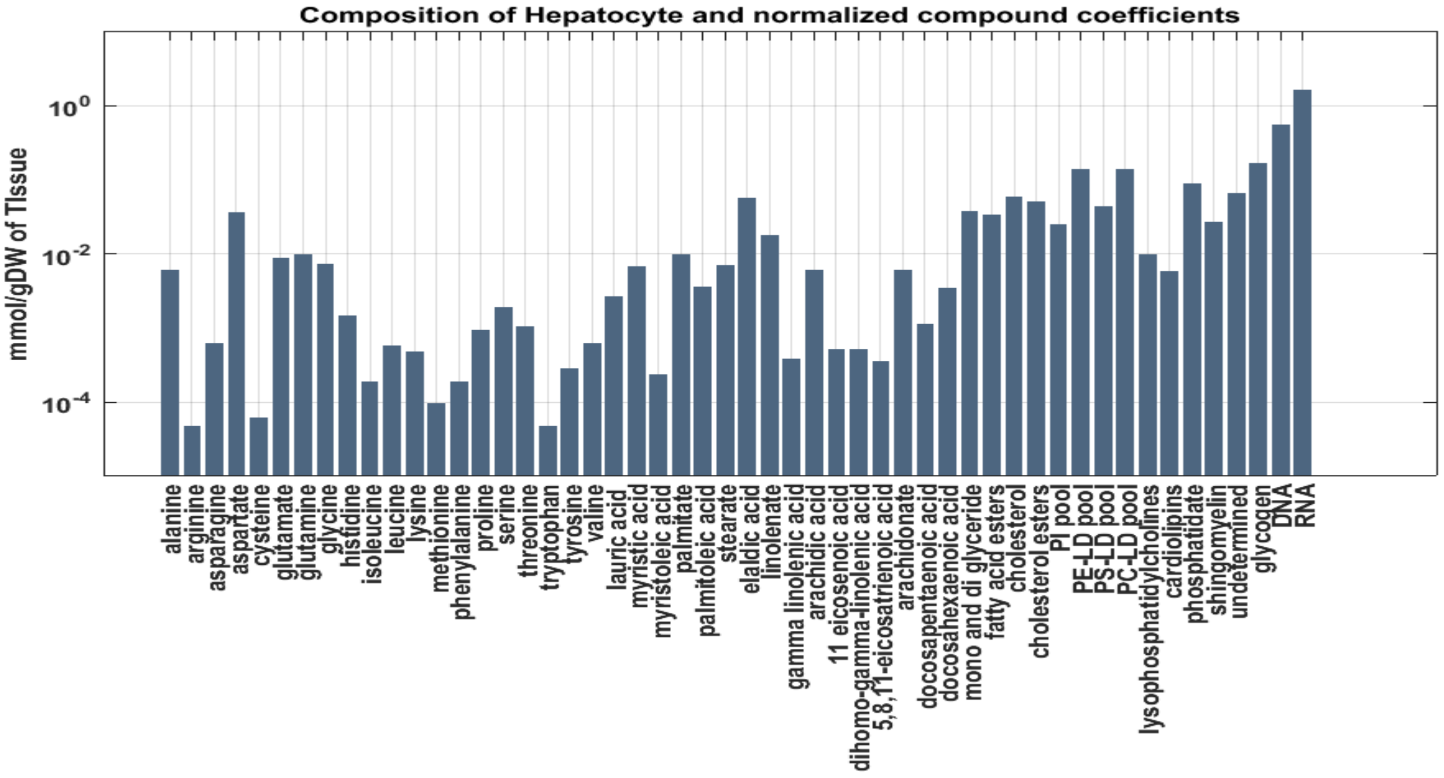

Fig.8. Bar plot of growth coefficients of hepatocytes or the molecular content in mmol/gDW/Tissue normalized for water contents. PC-LD, PE-LD,PS-LD pool denotes phosphatidyl choline, phosphatidyl ethanolamine and phosphatidyl serine pools respectively.

References

1 Bordbar, A. *et al.* A multi-tissue type genome-scale metabolic network for analysis of whole-body systems physiology. *BMC systems biology* **5**, 180 (2011).

2 Forbes, R., Cooper, A. & Mitchell, H. The composition of the adult human body as determined by chemical analysis. *Journal of Biological Chemistry* **203**, 359-366 (1953).

3 Thomas, L. W. The chemical composition of adipose tissue of man and mice. *Quarterly journal of experimental physiology and cognate medical sciences* **47**, 179-188 (1962).

4 Entenman, C., Goldwater, W. H., Ayres, N. S. & Behnke, A. R. Analysis of adipose tissue in relation to body weight loss in man. *Journal of applied physiology* **13**, 129-134 (1958).

5 BAKER, G. L. Human adipose tissue composition and age. *The American journal of clinical nutrition* **22**, 829-835 (1969).

6 Davidson, S. & Passmore, R. Human nutrition and dietetics. *Human nutrition and dietetics.* (1963).

7 Sanjurjo, P. *et al.* Fatty acid composition of skeletal muscle and adipose tissue in Spanish infants and children. *British journal of nutrition* **95**, 168-173 (2006).

8 Kabir, N. & Forsum, E. Estimation of total body fat and subcutaneous adipose tissue in full-term infants less than 3 months old. *Pediatric research* **34**, 448-454 (1993).

9 Wells, J. C. Toward body composition reference data for infants, children, and adolescents. *Advances in Nutrition: An International Review Journal* **5**, 320S-329S (2014).

10 Carberry, A. E., Colditz, P. B. & Lingwood, B. E. Body composition from birth to 4.5 months in infants born to non-obese women. *Pediatric research* **68**, 84-88 (2010).

11 Forster, J., Famili, I., Fu, P., Palsson, B. O. & Nielsen, J. Genome-scale reconstruction of the Saccharomyces cerevisiae metabolic network. *Genome Res* **13**, 244-253, doi:10.1101/gr.234503 (2003).

12 Heymsfield, S. B. *et al.* Biochemical composition of muscle in normal and semistarved human subjects: relevance to anthropometric measurements. *The American journal of clinical nutrition* **36**, 131-142 (1982).

13 Pouw, E. M., Schols, A. M., Deutz, N. E. & Wouters, E. F. Plasma and muscle amino acid levels in relation to resting energy expenditure and inflammation in stable chronic obstructive pulmonary disease. *American journal of respiratory and critical care medicine* **158**, 797-801 (1998).

14 Aas, V., Kase, E. T., Solberg, R., Jensen, J. & Rustan, A. C. Chronic hyperglycaemia promotes lipogenesis and triacylglycerol accumulation in human skeletal muscle cells. *Diabetologia* **47**, 1452-1461, doi:10.1007/s00125-004-1465-9 (2004).

15 Andersson, A., Sjödin, A., Hedman, A., Olsson, R. & Vessby, B. Fatty acid profile of skeletal muscle phospholipids in trained and untrained young men. *American Journal of Physiology-Endocrinology And Metabolism* **279**, E744-E751 (2000).

16 Bruce, Å. Skeletal muscle lipids. II. Changes in phospholipid composition in man from fetal to middle age. *Journal of lipid research* **15**, 103-108 (1974).

17 Sheikh, K., Förster, J. & Nielsen, L. K. Modeling hybridoma cell metabolism using a generic genome-scale metabolic model of Mus musculus. *Biotechnology progress* **21**, 112-121 (2005).

18 Shulman, R. J., Fiorotto, M. L., Sheng, H.-P., Finegold, M. J. & Garza, C. Liver composition and histology in growing infant miniature pigs given different total parenteral nutrition fuel mixes. *Journal of Parenteral and Enteral Nutrition* **11**, 275-279 (1987).

19 Pryce, J. W. *et al.* Reference ranges for organ weights of infants at autopsy: results of> 1,000 consecutive cases from a single centre. *BMC clinical pathology* **14**, 1 (2014).

20 Chan, S. C. *et al.* Estimating liver weight of adults by body weight and gender. *World journal of gastroenterology* **12**, 2217 (2006).
